# Resistance in the edges: a relational framework for treatment resistance in genitourinary cancers

**DOI:** 10.3389/fonc.2026.1974982

**Published:** 2026-10-12

**Authors:** Daniele Silva Lacerda, Rosiane Batista Mastelari, Tiago Veiras Collares

**Affiliations:** 1 University Hospital, Federal University of Pelotas - HUBrasil, Pelotas, Brazil; 2 Postgraduate Program in Biotechnology, Center for Technological Development, Federal University of Pelotas, Pelotas, Brazil

**Keywords:** cell–cell communication, genitourinary cancer, spatial oncology, treatment resistance, tumor microenvironment

## Abstract

Treatment resistance is usually attributed to genetic or adaptive changes within tumor cells. Here, we propose that, in some tumors, resistance also depends on specific interactions between malignant and non-malignant cells. We define relational resistance as a resistant phenotype that requires the functional organization of particular intercellular relationships. This concept is narrower than microenvironment-mediated resistance: the presence of stromal, immune, or vascular cells is not sufficient to establish relational resistance; a defined interaction must contribute functionally to treatment failure. We organize evidence along a five-step sequence: predicted edge, spatial support, functional edge, relational dependency, and therapeutic vulnerability. Genitourinary cancers illustrate different positions along this sequence: bladder cancer provides a predicted CD6–ALCAM edge with independent clinical association but without targeted perturbation; prostate cancer provides myeloid circuits with preclinical causal support and phase 1 proof-of-concept targetability; and renal cell carcinoma provides spatially organized multicellular programs with functional and preclinical perturbation support. We further propose relational memory as the hypothesis that a treatment-induced or treatment-selected interaction persists after therapy and influences response to a subsequent treatment. The framework therefore shifts the therapeutic question from which resistant cells are present to which relationships are required for resistance to persist.

## 1 Introduction: from resistant cells to resistant relationships

Treatment resistance is a dynamic evolutionary problem. Genetic selection, reversible drug-tolerant states, epigenetic adaptation, and lineage plasticity can all permit malignant cells to survive treatment (1–4). A complementary literature has shown that non-malignant compartments can protect or reprogram cancer cells, creating environment-mediated resistance and residual disease (5). Treatment resistance is therefore not exclusively cell-autonomous: therapy acts on a tumor ecosystem whose composition, spatial organization, and signaling relationships can change together. Recent systems-biology work likewise frames cancer stemness and therapy resistance as dynamic attractor states shaped by regulatory-network rewiring, plasticity, and microenvironmental stabilization (6).

Recent single-cell and spatial studies make this shift particularly visible. Pan-tissue analyses have identified coordinated multicellular modules that are rewired during cancer progression (7), while spatial ecotypes defined across millions of single-cell and spatial transcriptomes carry clinically relevant information, including associations with immunotherapy response (8). In another treatment setting, cellular communities in triple-negative breast cancer were associated with response to neoadjuvant chemotherapy (9). These studies support the importance of tissue-level organization; they do not, however, establish that every inferred interaction is causal or required for resistance.

This Perspective therefore advances a narrower proposition. We argue that some resistant phenotypes may be stabilized not only by the states of individual cells, but by the relationships connecting those states. The relational framework is intended to extend—not replace—clonal, epigenetic, microenvironmental, ecological, and evolutionary models of resistance. It is also complementary to systems-level attractor-state models: rather than treating ecosystem organization itself as sufficient evidence of resistance, it asks whether a specified intercellular edge is required for the resistant phenotype to persist. Its central question is simple: once the relevant cells have been identified, which relationships among them are required for the resistant phenotype to persist? Within this framework, relational resistance is the causal proposition that a specified intercellular interaction can become necessary for maintenance of the treatment-resistant phenotype.

## 2 Relational resistance: from nodes to dependencies

In network terms, malignant and non-malignant cell states can be treated as nodes, whereas their functional interactions are edges. An edge may represent ligand–receptor signaling, cytokine or chemokine exchange, metabolic coupling, extracellular-vesicle transfer, extracellular-matrix-dependent signaling, an immune synapse, a tunneling nanotube, or direct gap-junctional communication. This broad definition is deliberate: the relational framework is not tied to one molecular pathway, but to the biological consequence of an interaction between cellular states.

We define relational resistance as a treatment-resistant phenotype that depends, at least in part, on the organization and functional activity of intercellular relationships rather than exclusively on autonomous properties of the participating cells. This introduces relational heterogeneity: two tumors can contain similar cellular components yet differ in outcome because the interactions among those components differ. Put differently, composition is not topology, and topology is not function.

Relational resistance overlaps with, but is narrower than, the broader category of microenvironment-mediated resistance. The latter encompasses protective or adaptive effects arising from stromal, immune, vascular, matrix, and soluble components. The relational framework asks a more specific causal question: whether a defined interaction between specified cellular states is itself required to maintain resistance. Thus, the presence or abundance of a particular cell state can motivate a candidate mechanism but is insufficient to establish a relational dependency; perturbing the interaction must alter the resistant phenotype. Operationally, evidence for relational resistance requires showing that perturbation of a specified interaction changes treatment response after the participating cell states have been defined; abundance, co-occurrence, or microenvironmental association alone is insufficient.

The evidentiary threshold is crucial. CellChat, NicheNet, CellPhoneDB, and related tools can prioritize candidate ligand–receptor relationships (10–12), but computational probability is not mechanism. We therefore use a single five-step evidence ladder throughout this Perspective: (1) predicted edge, a computationally or molecularly inferred interaction; (2) spatial support, evidence that the participating cellular states occupy a compatible tissue context; (3) functional edge, experimental evidence that the interaction produces a biological effect; (4) relational dependency, treatment-context perturbation of the interaction that changes the resistant phenotype; and (5) therapeutic vulnerability, a relational dependency for which targetability has additional translational or clinical support. The levels are cumulative, and therapeutic vulnerability should not be assumed from dependency alone. **Figure 1** summarizes this progression within the transition from pretreatment ecosystem to resistant ecosystem. The ladder is hierarchical with respect to evidentiary support for a candidate edge, but its final step addresses a distinct translational question: levels 1–4 progressively establish inference, spatial compatibility, function, and treatment-context causal dependency, whereas level 5 adds evidence that the dependency can be targeted in a clinically relevant manner. Therapeutic vulnerability therefore should not be interpreted as greater causal certainty, and early clinical signals are labeled explicitly as such.

**Figure 1:**
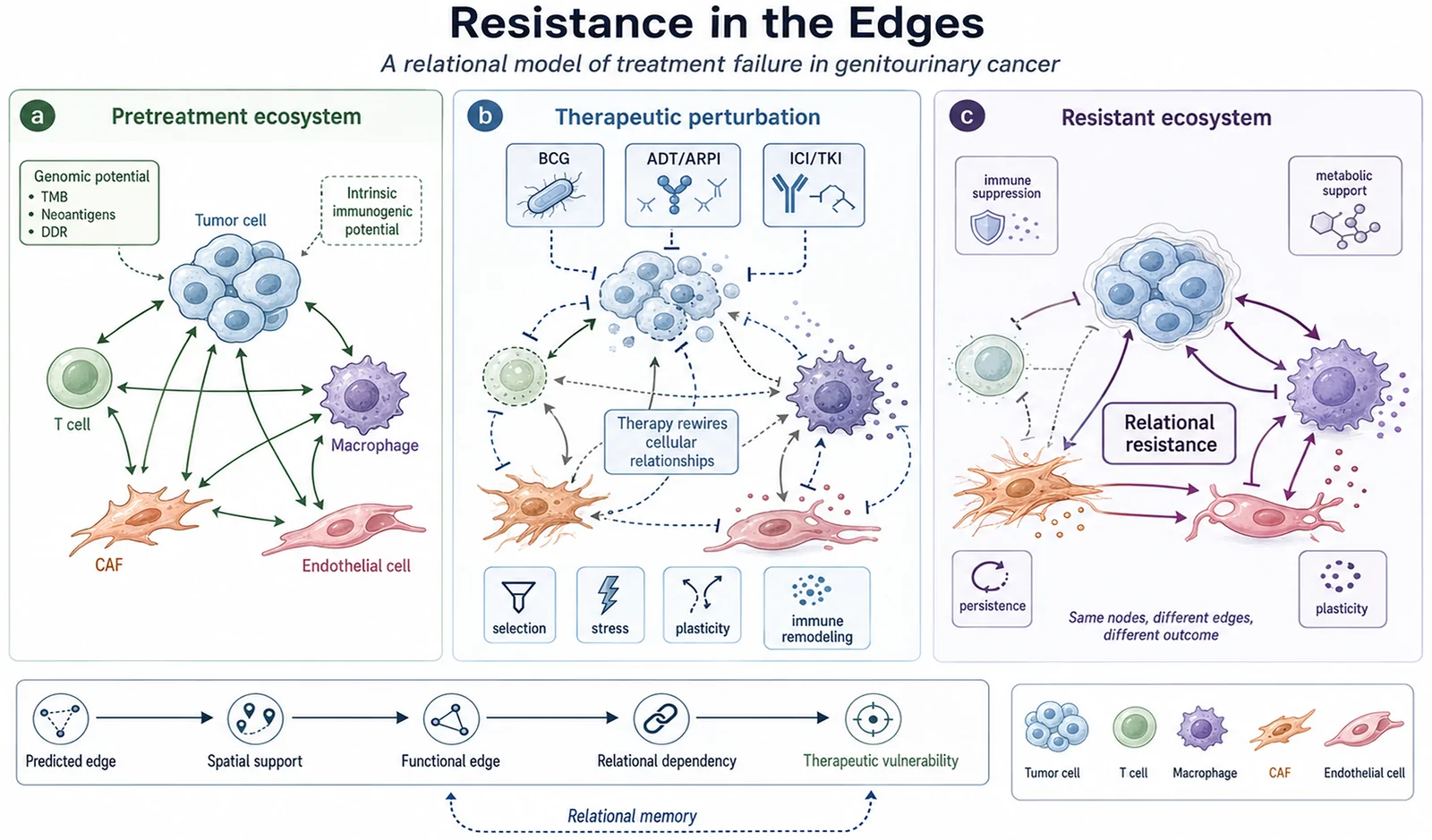
Relational framework of treatment resistance. Therapy acts on a pretreatment ecosystem and can reorganize interactions among malignant, immune, stromal, and vascular cells, yielding a resistant ecosystem with altered edge architecture. The evidence ladder progresses from predicted edge to spatial support, functional edge, relational dependency, and therapeutic vulnerability. Solid arrows indicate forward evidentiary progression; the dashed return arrow denotes the hypothesis of relational memory, in which a treatment-conditioned interaction pattern persists and influences a subsequent therapeutic response. Therapeutic vulnerability additionally requires clinically relevant targetability and separation from essential homeostatic communication.

Association, co-localization, or increased inferred communication can prioritize an edge for testing, but none alone establishes dependency. Association is not function; function is not dependency; and dependency is not automatically clinical actionability. Accordingly, ligand and receptor expression—even in compatible cell types—defines only a predicted edge unless functional signaling is demonstrated.

## 3 Genitourinary cancers as complementary test systems for relational resistance

Bladder, prostate, and renal cancers are exposed to biologically distinct therapeutic pressures, yet each provides evidence that treatment outcome cannot always be reduced to a single malignant-cell state. **Table 1** summarizes representative examples and, importantly, the level of evidence supporting each proposed relational mechanism.

**Table 1:** Representative relational mechanisms associated with treatment resistance in genitourinary cancers.

Setting	Representative relational edge(s)	Highest evidence-ladder level reached	Key relational question
NMIBC/BCG	CD6–ALCAM T-cell–urothelial signaling; epithelial/immune/stromal BCG-response subtypes (14, 15)	Predicted edge (independent clinical association)	Does selective perturbation of CD6–ALCAM alter the BCG-resistant phenotype?
Prostate/ARSI	Club-like epithelial–PMN-MDSC interaction (16)	Spatial support	Does this epithelial–myeloid interaction causally maintain persistence under androgen-axis therapy?
Prostate/ARSI	CXCR2-dependent myeloid chemotaxis (17)	Therapeutic vulnerability (phase 1 proof-of-concept signal)	Can CXCR2 blockade reproducibly restore ARSI sensitivity in larger controlled cohorts?
Prostate/ICI	SPP1-high TAM–adenosine suppression of CD8 T cells (18)	Therapeutic vulnerability (phase 1 signal)	Can A2A receptor blockade overcome ICI resistance in prospectively selected patients?
ccRCC/ICI	CXCL14 tumor–APOE TAM–endothelial program (19)	Functional edge (with pathway-level preclinical perturbation)	Does selective perturbation of the CXCL14-associated tumor–TAM interaction, rather than broader CXCR4 blockade, reverse ICI resistance?
ccRCC/ICI–TKI	SAA tumor–CEACAM1+ neutrophil–TIM-3+ T-cell circuit (20)	Relational dependency (preclinical *in vivo*)	Can selective targeting of the SAA or CEACAM1–TIM-3 components improve ICI–TKI efficacy in patients?
Junctional communication	Connexin/gap-junction coupling as a physically instantiated edge; context-dependent channel and non-channel functions (21–30)	Functional edge exemplar; treatment-specific dependency not established	Which defined cell pair, transferred cargo, and connexin function becomes a treatment-specific relational dependency?

Evidence classifications are intentionally conservative. Qualifiers such as “phase 1,” “preclinical,” and “treatment-specific dependency not established” indicate the boundary between mechanistic support, early targetability signals, and validated clinical efficacy.

### 3.1 Bladder cancer: genomic potential and relational realization

Non-muscle-invasive bladder cancer (NMIBC) is particularly instructive because intravesical BCG requires successful coordination between tumor immunogenicity and host immunity. Bastos et al. showed that pretreatment tumors with higher tumor mutational burden and predicted neoantigen load were more likely to respond to BCG and had longer recurrence-free survival; deleterious DNA-damage-response alterations were also associated with improved recurrence-free survival (13). These results support the idea that tumor antigenicity contributes to intrinsic immunogenic potential, but the study also acknowledged that tumor-microenvironmental and host-immune determinants were not captured.

That gap has become increasingly important. Molecular stratification of high-risk NMIBC identified BCG-response subtypes with distinct epithelial, immune, and stromal features, including an immunosuppressive subtype enriched after recurrence (14). More recently, single-cell analysis of BCG-naïve and recurrent NMIBC found broadly similar genomic alterations and cellular composition but a marked increase in CD6–ALCAM interactions between T cells and urothelial cells in recurrent tumors. CD6-high T cells showed reduced activation-associated programs, and a CD6/ALCAM signature was associated with shorter recurrence-free survival in an independent cohort (15).

Together, these studies motivate the distinction between genomic potential and relational realization. Here, relational realization denotes the conversion of tumor-intrinsic therapeutic potential into treatment outcome through a specific, functionally consequential pattern of intercellular interactions. Neoantigenicity can create an opportunity for immune control, but that opportunity must be executed through antigen presentation, immune recruitment, recognition, activation, and effector contact. BCG failure may therefore arise even when tumor-intrinsic immunogenicity is favorable if the relationships needed to translate that potential into tumor killing are absent, dysfunctional, or redirected toward suppressive configurations. The Juric et al. dataset provides a concrete example of the type of incremental information sought by the framework: genomic alterations and overall cellular composition were broadly similar, whereas inferred CD6–ALCAM signaling differed in recurrent tumors and the combined signature was associated with shorter recurrence-free survival in an independent cohort (15). Because targeted perturbation was not performed, however, CD6–ALCAM is best classified as a predicted edge with clinical association rather than a demonstrated relational dependency.

### 3.2 Prostate cancer: resistant states embedded in myeloid circuits

Prostate cancer tests the framework under androgen receptor signaling inhibitor (ARSI) pressure. Kiviaho et al. combined single-cell and spatial transcriptomics across the treatment continuum and identified club-like epithelial cells as an interface between the epithelium and immune microenvironment. Club-like regions showed reduced androgen signaling, luminal-progenitor features, a senescence-associated secretory phenotype, and association with polymorphonuclear myeloid-derived suppressor cell activity (16). The relational interpretation is not simply that club-like cells are resistant; rather, their persistence may become consequential through the myeloid environment they recruit or sustain.

Functional and early clinical evidence supports specific myeloid circuits, but these mechanisms should be considered separately. Guo et al. showed that senescence-associated CXCR2-ligand production contributes to myeloid recruitment and resistance to androgen receptor pathway inhibition. In a phase 1 proof-of-concept study enrolling 23 patients with metastatic castration-resistant prostate cancer that had progressed on androgen receptor signaling inhibitors, CXCR2 inhibition with AZD5069 plus enzalutamide decreased circulating neutrophil levels, reduced intratumoral suppressive myeloid infiltration, and produced biochemical and radiological responses in a subset of patients (17). These findings provide an early clinical targetability signal for CXCR2-dependent myeloid recruitment, but the small, non-randomized phase 1 study does not establish general clinical validation of myeloid-mediated resistance. Separately, Lyu et al. identified SPP1-high tumor-associated macrophages that accumulate with disease progression, suppress CD8 T-cell activity through adenosine signaling, and promote resistance to immune checkpoint inhibition. Pharmacologic A2A receptor inhibition reversed this phenotype in experimental models and, in combination with atezolizumab, produced clinical responses in a subset of patients with metastatic castration-resistant prostate cancer (18). This represents a distinct SPP1-high-TAM–adenosine–CD8 T-cell resistance circuit and an early clinical signal rather than established therapeutic validation.

### 3.3 Renal cancer: from multicellular programs to perturbable edges

Clear-cell renal cell carcinoma (ccRCC) provides a particularly informative preclinical example of progression along the evidence ladder. Yao et al. integrated single-cell and spatial transcriptomics and identified an immune-checkpoint-blockade-resistant program involving CXCL14-expressing malignant cells, APOE-expressing tumor-associated macrophages, and endothelial cells. In this program, CXCL14-expressing tumor cells were linked to recruitment of APOE-expressing macrophages and subsequent metabolic and angiogenic remodeling. The CXCL14–CXCR4 relationship was inferred as part of the communication analysis, and the study further reported functional validation of the CXCL14–TAM interaction together with a preclinical dual CXCR4/PD-1 perturbation that restored CD8 T-cell cytotoxicity and reversed resistant features (19). Because CXCR4 blockade perturbs a broader chemokine pathway and does not selectively isolate the contribution of the CXCL14-associated tumor–macrophage–endothelial program, we classify this example conservatively as a functional edge with pathway-level preclinical perturbation support rather than definitive proof that the full multicellular program is a treatment-specific relational dependency.

An independent treatment-linked study of pre- and post-treatment advanced RCC samples under TKI alone or TKI plus PD-1 blockade identified treatment-associated enrichment of SAA-positive tumor cells and an immunosuppressive VEGFA-positive/CEACAM1-positive neutrophil population in non-responders. Pharmacologic SAA inhibition increased sensitivity to combined therapy, and disruption of the CEACAM1–TIM-3 interaction potentiated anti-PD-1 activity in humanized models (20). These findings reach the relational-dependency level of the same five-step ladder because they combine predicted and spatially supported interactions with functional evidence and treatment-context perturbation. They do not, by themselves, establish therapeutic vulnerability, which requires additional evidence of clinically relevant targetability.

## 4 Connexins as a physical-edge exemplar within the relational architecture

Most examples above involve ligand–receptor or chemokine-mediated signaling. Connexins provide a complementary physical-edge exemplar because gap junctions directly couple neighboring cells and permit exchange of ions and small metabolites (21). This makes them useful for the relational framework not as a separate theory of resistance, but as a system in which an intercellular edge can be measured and experimentally perturbed.

Genitourinary studies also illustrate why connexin expression cannot be equated with a resistance edge. Highly aggressive prostate cancer cells can retain connexin expression while lacking canonical channel activity (28); GJB3 can influence cytoskeletal organization, chromosomal stability, and invasion through channel-independent interactions (29); and suppression of Cx32-mediated communication in differentiated urothelium induces a migratory TGF-β/SMAD- and extracellular-matrix-remodeling phenotype (30). These observations establish context-dependent function, but none alone demonstrates a treatment-specific relational dependency.

Accordingly, a translational strategy should not aim to restore or inhibit connexin communication indiscriminately. The relevant question is relational: which cells are connected, what is transferred or scaffolded, at what stage and treatment context, and does that interaction meet the evidentiary threshold of a resistance dependency? This framing keeps the focus on treatment-context-specific dependency rather than on connexin biology per se, which has been reviewed extensively elsewhere (26, 27).

## 5 Relational memory and the residual relational state

Relational memory is the most explicitly hypothesis-driven component of the framework. We define relational memory as the persistence of a treatment-induced or treatment-selected organization of intercellular relationships that influences the response of the residual tumor ecosystem to a subsequent therapeutic challenge. The evidence discussed above shows treatment-associated remodeling; it does not yet establish a generalized multicellular memory. We therefore present relational memory as a hypothesis, not a demonstrated hallmark.

Operationally, relational memory should be distinguished from transient treatment-induced remodeling. Demonstrating relational memory would require evidence that a treatment-induced or treatment-selected interaction pattern persists beyond the initial therapeutic perturbation and modifies the response to a subsequent therapeutic challenge. Stronger evidence would additionally require showing that disruption of the persistent interaction attenuates or abolishes this history-dependent effect. Thus, treatment-associated remodeling alone would not constitute relational memory unless the remodeled architecture persists and carries functional consequences for subsequent response. Persistent microenvironmental remodeling alone is therefore insufficient: added value requires a defined interaction that carries treatment history into a later response and, ideally, loss of that history-dependent effect when the persistent edge is perturbed.

The hypothesis extends established observations that treatment history can persist through genetic selection, chromatin-mediated drug tolerance, and environmentally supported residual disease (2–5). A post-treatment tumor may have low malignant burden yet retain remodeled fibroblasts, suppressive myeloid populations, dysfunctional lymphocytes, altered vasculature, extracellular matrix, or direct cell-coupling states. Thus, a subsequent therapy may encounter an ecosystem partly conditioned by the preceding treatment. The lower loop in **Figure 1** represents this history dependence.

This reasoning also motivates a relational residual state: a post-treatment condition in which potentially resistance-permissive relationships persist even when malignant-cell burden is low. We deliberately avoid the term ‘relational residual disease,’ because current evidence does not justify a new clinical entity. The testable prediction is narrower: relational divergence may become detectable before overt clonal recurrence and, if independently validated, may add information to risk assessment beyond genotype and cell-state composition. Operationally, this differs from minimal residual disease because it refers to persistence of interaction architecture rather than residual malignant-cell burden; it does not imply a new clinical disease category.

## 6 Scope, boundary conditions, and testability of the relational framework

The relational framework is intended as a causal refinement of microenvironment-mediated resistance, not as a replacement for cell-autonomous mechanisms. It applies only when a defined interaction between specified cellular states contributes to a resistant phenotype. Increased communication, cellular proximity, or ligand–receptor co-expression alone is therefore insufficient to establish relational resistance. These observations identify candidate edges, but a relational dependency requires functional perturbation under the relevant treatment context that changes the resistant phenotype. The framework also does not assume that intercellular communication is intrinsically detrimental: productive immune interactions may promote tumor elimination, whereas suppressive, trophic, or metabolic interactions may support treatment failure. The biological consequence of an edge therefore depends on the participating cellular states, the direction and content of signaling, and the treatment context. The framework therefore has added value only if edge-level measurements or perturbations explain mechanism or treatment response after accounting for genotype, cell state, cellular composition, and the broader ecological context.

A further limitation concerns sampling. Tumor ecosystems are spatially and temporally heterogeneous, and a single biopsy cannot establish persistence, temporal precedence, or relational memory. Multicellular modules and spatial ecotypes can be reproducible and clinically informative (7–9), but association is not equivalent to causal necessity. These concepts therefore require longitudinal sampling and, ultimately, experimental perturbation. The framework is experimentally testable: if relational measurements or perturbations add no mechanistic or predictive information beyond genotype, cellular-state composition, and established microenvironmental features, the framework has limited added value. Its purpose is to generate perturbable hypotheses, not simply to rename the tumor microenvironment as a network.

Two examples illustrate the intended incremental value without overstating causality. In NMIBC, inferred CD6–ALCAM signaling differentiated BCG-recurrent from BCG-naïve tumors despite broadly similar genomic alterations and cellular composition (15). In ccRCC, a spatially organized multicellular program associated with ICB resistance contributed tissue-organization information beyond cell identity alone, although the causal specificity of individual edges remains incompletely isolated (19).

## 7 Translational and experimental agenda

Testing relational resistance—and particularly relational memory—requires longitudinal designs. Pretreatment sampling should be followed, where feasible, by early-treatment, maximal-response, residual-state, and recurrence sampling. Genomics can define clonal evolution; single-cell analysis can define cellular states; spatial transcriptomics or proteomics can establish compatible tissue organization; and communication inference can prioritize candidate edges. The decisive evidentiary step is functional perturbation—blocking a ligand–receptor pair, editing a sender or receiver program, disrupting direct coupling, or reconstructing the interaction in organoid–immune, stromal co-culture, or *in vivo* models.

Serial tissue biopsies will often be impractical or ethically unjustified. Longitudinal studies can therefore combine clinically indicated tissue sampling with liquid-biopsy approaches, including circulating tumor DNA, circulating tumor cells, circulating immune-cell phenotyping, and soluble mediators, as well as serial molecular or functional imaging were informative. These approaches can establish temporal change or provide relational proxies, but they cannot by themselves prove spatially organized or functional edges; tissue-based spatial imaging and experimental perturbation remain necessary for the higher levels of the evidence ladder.

Added predictive value should be tested using prespecified nested models in which relational features are added to baseline models containing genotype, cell-state abundance, clinical covariates, and established microenvironmental variables. Improvement should be assessed out of sample using appropriate measures of discrimination and calibration (for example, time-to-event concordance or area under the curve where applicable), supported by likelihood-based model comparison and independent validation rather than by in-sample fit alone.

For relational memory, longitudinal designs should additionally distinguish transient remodeling from persistent interaction states and determine whether persistence alters the response to a subsequent therapeutic challenge. Where feasible, perturbation of the persistent edge should test whether the history-dependent effect itself can be attenuated or abolished.

The relational framework yields five testable predictions. Each specifies a result that would support added explanatory value beyond established cell-state and microenvironmental frameworks and, conversely, a result whose absence would weaken the framework:

1. Temporal precedence. If relational remodeling contributes to the emergence of resistance, changes in defined intercellular edges should become detectable during treatment before overt clonal recurrence in at least a subset of tumors. This can be tested by longitudinally comparing the timing and predictive value of relational changes with genomic and cell-state evolution.
2. Architecture beyond composition. Tumors with similar genomic alterations and broadly similar cellular composition may show different treatment responses when the functional interactions among those cells differ. This prediction requires showing that relational architecture adds predictive information after accounting for genotype and cell abundance.
3. Convergent relational function. Different cancers or treatments may use molecularly distinct edges to generate analogous resistance-supporting functions, such as myeloid recruitment, T-cell suppression, metabolic support, or vascular remodeling. Cross-tumor and cross-treatment analyses should therefore reveal functional convergence even when the molecular interactions differ. A concrete test would compare molecularly distinct recruitment circuits—for example, CXCR2-dependent myeloid recruitment in ARSI-treated prostate cancer and CXCL14-associated TAM recruitment in ccRCC—and ask whether perturbing each edge produces a convergent downstream phenotype, such as reduced suppressive myeloid activity, restored CD8 T-cell function, and improved treatment response.
4. Causal reversibility. An interaction that reaches the level of relational dependency should be causally perturbable: disrupting that interaction under the relevant treatment context should reduce resistance or restore therapeutic sensitivity. This is the central experimental criterion distinguishing dependency from association.
5. History dependence and relational memory. If relational memory exists, an interaction architecture induced or selected by a preceding therapy should persist sufficiently long to modify the response to a subsequent therapy. The strongest test would show that disruption of the persistent interaction attenuates or abolishes this history-dependent effect.

If these predictions are not supported in adequately powered longitudinal and perturbation studies, the relational framework would have limited added explanatory or predictive value beyond established cell-state and microenvironmental frameworks.

### 7.1 Limitations and paths to validation

As a Perspective, this framework is hypothesis-generating, and its principal limitations define how the framework should be tested and refined rather than shortcomings that can be resolved within the present article.

- Conceptual overlap. Relational measurements may overlap with established cell-state and microenvironmental variables. This limitation should be addressed by testing whether edge-level measurements add mechanistic or predictive information after accounting for genotype, cell abundance, and established microenvironmental features.
- Association versus causality. Inferred communication and spatial proximity do not establish a relational dependency. Candidate edges should therefore progress through the evidence ladder, with treatment-context perturbation required before an interaction is classified as a causal dependency (10–12).
- Spatial and temporal sampling. Tumor ecosystems are heterogeneous, and single biopsies may miss transient, spatially restricted, or persistent interactions. Longitudinal and, where feasible, multisite sampling will be required to distinguish treatment-associated remodeling from relational memory (7–9).
- Network complexity and model overfitting. The number of possible intercellular interactions creates risks of confounding, multiple testing, and overinterpretation. Candidate edges should therefore be prespecified where possible from treatment biology, prioritized using explicit biological criteria, analyzed with dimension-reduction or regularized approaches when high-dimensional discovery is unavoidable, and validated in independent cohorts and complementary experimental systems.
- Dependency versus therapeutic targetability. An experimentally perturbable edge is not necessarily a safe or clinically actionable target. Translational development should therefore demonstrate that the resistance-supporting interaction can be disrupted with sufficient specificity and without broadly impairing essential homeostatic communication.

Together, these limitations establish practical criteria for testing, refining, or potentially rejecting the relational framework while preserving its intended role as a conceptual framework that extends, rather than replaces, established models of treatment resistance.

The translational objective is therefore not to inhibit communication broadly, but to identify a dependency that is both mechanistically necessary and therapeutically separable from homeostatic communication. **Table 1** makes this distinction explicit across the principal genitourinary examples. If validated prospectively, relational biomarkers could complement molecular biomarkers by reporting not only what the tumor contains, but how the components are functionally organized at the moment a treatment decision is made.

## 8 Discussion and outlook

The relational framework does not replace genomic, epigenetic, evolutionary, ecological, or cell-state explanations of resistance; it adds a complementary level of organization. Cancer genomics identifies what has changed within cells. Single-cell analysis identifies which cellular states are present. Spatial biology defines where those states reside. Systems-level attractor models emphasize dynamic state transitions and network stabilization (6). A relational approach asks the narrower causal question of which interactions among those states are required for resistance to persist.

Genitourinary cancers are well suited to test this idea because BCG, androgen-axis therapy, and immune/antiangiogenic combinations perturb very different tissues yet repeatedly expose resistance-supporting interactions. The value of the framework will depend on demonstrating that relational measurements or perturbations add mechanistic or predictive information beyond genotype, cell-state composition, and broader microenvironmental features. The next advance may therefore depend not only on resolving resistant cells at ever greater molecular depth, but on determining why particular cells must remain connected for resistance to survive. If resistant tumors are partly stabilized by relationships, some of their most actionable vulnerabilities may lie not within the nodes, but in the edges between them.
